# Short-Term Effects of Anterior and Posterior Bite Blocks on the Electromyographic Activity of the Masticatory Muscles in Healthy Volunteers: An Analysis of Variability

**DOI:** 10.3390/dj14080465

**Published:** 2026-07-30

**Authors:** Vincenzo D’Antò, Giorgio Oliva, Rosaria Bucci, Roberto Rongo, Ruggiero Valentino, Ambrosina Michelotti

**Affiliations:** Section of Orthodontics, Department of Neuroscience, Reproductive Sciences and Oral Sciences, University of Naples Federico II, 80131 Naples, Italy; vincenzo.danto@unina.it (V.D.); rosaria.bucci@unina.it (R.B.); roberto.rongo@unina.it (R.R.); ru.valentino@studenti.unina.it (R.V.); michelot@unina.it (A.M.)

**Keywords:** bite blocks, clear aligners, surface electromyography, masticatory muscles, somatosensory amplification, cluster analysis, orthodontics

## Abstract

**Background/Objectives:** Bite blocks (BBs) are commonly used in clear aligner therapy, yet their effect on masticatory muscle activity is poorly characterised. This study aims to examine the variability in the effects of anterior and posterior BBs on masticatory muscle activity in healthy participants. **Methods:** Twenty-four healthy adults were included in a crossover design comparing anterior and posterior BBs in removable passive aligners. Surface electromyography measured muscle activity at baseline, 24 h, 48 h and one week. Participants completed psychosocial questionnaires. Response patterns were identified via cluster analysis, and psychosocial differences were assessed with *t*-tests. **Results:** Anterior BBs immediately reduced muscle work intensity, with significant inter-individual variability (*p* = 0.030 at T1; *p* = 0.047 at T3). In contrast, posterior BBs altered muscle activity without significant variability over time (*p* > 0.05). Cluster analysis applied to anterior BB revealed two distinct adaptation patterns, which had significantly different Somatosensory Amplification Scale scores (*p* = 0.040). **Conclusions:** Within the limitations of the study, in the same participants, wearing passive aligners with anterior bite blocks significantly increased the inter-individual variability of muscular work intensity relative to the no-appliance baseline, whereas passive aligners with posterior bite blocks produced no significant change in variability.

## 1. Introduction

Orthodontic bite turbos are artificial surfaces that prevent complete jaw closure, also referred to as bite blocks (BB), bite planes or bite ramps [1]. They are employed in orthodontics for various clinical purposes, including bracket protection, malocclusion correction and anchorage reinforcement [2,3,4,5]. By modifying the occlusal scheme and temporarily increasing the vertical dimension, BBs facilitate specific tooth movements and can improve the biomechanical efficiency of orthodontic treatment. Their use has expanded with the introduction of clear aligner therapy, where digitally planned bite ramps are incorporated into the aligners to achieve similar therapeutic objectives. Clear aligner therapy has become an increasingly popular alternative to fixed orthodontic appliances owing to its aesthetics, comfort and digital workflow [6]. Integrated bite ramps are frequently used for deep bite correction, while posterior bite blocks may assist vertical control and contribute to the management of anterior open bite malocclusions [7,8,9]. Despite their widespread clinical use, the biological and functional consequences of these occlusal modifications remain incompletely understood. Changes in occlusal contacts and vertical dimension can influence the neuromuscular balance of the stomatognathic system. Previous investigations demonstrated that the number and distribution of occlusal contacts are associated with masticatory muscle activity in healthy individuals [10]. Similarly, experimental increases in vertical dimension have been shown to affect the electromyographic activity of the masseter and temporalis muscles as well as maximum bite force [11,12,13,14,15]. These findings suggest that appliances introducing artificial occlusal contacts may induce adaptive neuromuscular responses aimed at restoring functional equilibrium. Bite blocks may therefore act not only as biomechanical auxiliaries for tooth movement but also as transient functional stimuli capable of eliciting neuromuscular adaptation. However, the magnitude and direction of these responses appear to vary substantially among individuals, suggesting the involvement of patient-specific adaptive mechanisms [16,17,18]. Surface electromyography (sEMG) is a widely accepted, non-invasive method for assessing the functional status of masticatory muscles. It has been extensively used to evaluate muscular adaptations induced by orthodontic and prosthodontic appliances, including occlusal splints [11,12,13,19,20,21,22], bite planes and bite blocks [16,17,18], as well as clear aligners [23,24,25,26,27,28]. However, the reported effects of these appliances on muscular activity are inconsistent. Studies investigating occlusal splints have shown both increases and decreases in electromyographic activity depending on appliance characteristics and assessment conditions [11,12,13,19,20,21]. Likewise, investigations into orthodontic bite-raising devices have reported heterogeneous outcomes, ranging from enhanced muscular recruitment to reduced activity or negligible changes [16,17,18]. A similar variability has been observed in studies evaluating clear aligner therapy. Some authors reported transient increases in masticatory muscle activity immediately after aligner insertion [23,27], whereas others found reductions or no significant alterations over time [24,25,28]. Recent systematic reviews and meta-analyses concluded that current evidence is insufficient to establish a consistent effect of clear aligners on masticatory muscle function [29,30]. Furthermore, individual factors such as oral parafunctional behaviours may contribute substantially to the observed inter-subject variability [26,31]. Although bite ramps and bite blocks are routinely integrated into contemporary aligner therapy, no previous study has specifically evaluated their influence on masticatory muscle activity when combined with aligners. Since these devices simultaneously modify occlusal contacts and vertical dimension, their neuromuscular effects may differ from those induced by aligners alone. Understanding the variability of these responses may provide useful information regarding patient adaptation and the functional implications of aligner-based bite-raising protocols. Therefore, the aim of the present study was to investigate the variability of the muscular response induced by bite blocks incorporated into passive aligners in a sample of healthy adults. The null hypothesis was that wearing passive aligners incorporating anterior or posterior bite blocks would not increase the inter-individual variability of masticatory muscle activity.

## 2. Materials and Methods

This study was designed as a randomised crossover experimental investigation aimed at evaluating the neuromuscular response to different configurations of passive clear aligners with integrated bite blocks. Healthy adult participants were enrolled and underwent standardised electromyographic (sEMG) recordings of the masticatory muscles under controlled occlusal conditions. Each participant was exposed to two experimental passive clear aligner (PCA) configurations in a crossover design. The study protocol included two groups:

- Group 1: Delivery of PCA with anterior Bite Blocks –> one week of washout period –> Delivery of PCA with posterior Bite Blocks.
- Group 2: Delivery of PCA with posterior Bite Blocks –> one week of washout period –> Delivery of PCA with anterior Bite Blocks.

A washout period was necessary to minimise carryover effects between experimental phases. Electromyographic activity was recorded at baseline and at multiple time points following appliance delivery in order to assess both immediate and short-term neuromuscular adaptations. Standardised functional tests, including maximum voluntary clenching with and without occlusal support, were performed to evaluate changes in muscle recruitment patterns under different occlusal conditions. In addition, psychological and behavioural questionnaires were administered to explore potential individual factors associated with neuromuscular variability. All recordings were performed using a standardised protocol to ensure the repeatability and comparability of electromyographic data across conditions and time points. Statistical analyses were then conducted to assess changes in variability of muscular response and to identify potential subgroups with distinct neuromuscular adaptation patterns.

### 2.1. Sample

The sample size was calculated based on data from Chandu and colleagues [13]. A variance ratio of roughly 2.5 was observed and considered clinically relevant. Using a sample size calculation based on the log transformation of the variance ratio, with α = 0.05 (two-tailed) and β = 0.20 (power = 0.80), the estimated sample size was approximately 20 subjects. A sample of 24 healthy volunteers (6 males, 18 females; mean age 27.28 ± 3.77 years) was recruited from the staff and students of the Faculty of Dentistry at the University of Naples Federico II (Italy).

Inclusion criteria were adults (>18 years) with full permanent dentition (excluding the third molars) and more than 10 occlusal contacts [10]. Exclusion criteria included a presence or history of orofacial pain, ongoing orthodontic treatment and regular use of medications.

### 2.2. Appliances and Configurations

Direct intraoral scans were taken using the iTero Element 5D Plus system (Align Technologies). A single trained operator acquired the impressions and immediately verified that the STL file met the success criteria: clearly defined marginal contours and no scan holes.

The STL files were sent to a dental laboratory specialising in orthodontic appliance manufacturing. The following different configurations of the passive clear aligner (PCA) were produced:

One upper PCA with an anterior BB, extending from 1.2 to 2.2. The BB was 2 mm high, and its length was constructed in accordance with the patient’s overjet to ensure equally distributed dental contact in the anterior region.

One lower PCA with posterior BBs, positioned on both the left and right, extending at 3.6–3.7 and at 4.6–4.7. The BBs were 2 mm high.

One upper and one lower passive PCA, designed with the same material and thickness as the experimental PCAs, but without any BBs.

All PCAs were manufactured from a blend of thermoplastic materials with a thickness of 0.75 mm. The thermoplastic sheets used for the fabrication of the passive clear aligners were made of Duran^®^ material (SCHEU-DENTAL GmbH, Iserlohn, Germany). All passive clear aligners were manufactured in the in-house orthodontic laboratory by experienced dental technicians following standardised thermoforming procedures. This ensured consistency in appliance fabrication and adherence to the study design specifications.

Two configurations were produced:

Configuration 1: upper PCA with anterior BB + lower passive PCA;

Configuration 2: upper passive PCA + lower PCA with posterior BBs.

### 2.3. Questionnaires

Participants were invited to complete the following questionnaires:

SSAS—Somatosensory Amplification Scale [32]: to quantify an individual’s tendency toward somatosensory amplification;

OBC—Oral Behaviour Checklist [33]: to investigate the frequency of certain oral activities performed by the patient in the last month.

### 2.4. Electromyographic Examination

The electromyographic examination quantified the influence of tooth-periodontal proprioception on the recruitment of chewing muscles. The surface electromyography (sEMG) activity was recorded using a computerised instrument (Easymyo^®^, 3 Technology Ltd., Udine, Italy) following a standardised protocol. Skin preparation was performed prior to electrode placement in order to reduce skin impedance and improve signal quality. The skin over the masseter and anterior temporalis muscle regions was cleaned using alcohol wipes to remove surface oils and debris. A conductive gel was then applied to further reduce impedance and enhance electrode–skin contact. When necessary, gentle abrasion with sterile gauze was performed to ensure optimal signal acquisition.

Following skin preparation, bipolar surface electrodes were positioned over the masseter and anterior temporalis muscles according to Ferrario et al. [10]. The sEMG signal was amplified (CMRR 100 dB), digitised (24-bit, 4000 Hz) and digitally filtered (30–400 Hz bandpass with 50–60 Hz notch).

The sEMG activity was recorded during the following tests:

Maximum Voluntary Clenching (MVC): the subject was instructed to clench as hard as possible and maintain the contraction throughout the test (5 s).

Maximum Voluntary Clenching with Cotton Roll Interposition (COT): the subject was asked to clench in the same manner as the previous test (5 s), with a cotton roll (10 mm diameter) placed on each side between the arches. The test was repeated twice.

Tests were separated by ≥3 min rest periods to prevent muscle fatigue. The EMG protocol was identical with and without PCAs.

The electromyographic waves from each subject’s muscles were analysed, and the electromyographic graphs along with two static indices were calculated:

IMPACT is the ratio of muscle activity during a test versus a normalisation test. It reflects occlusal stability, showing that more and larger occlusal contacts improve muscle activity. Healthy subjects show 80–120% of the activity recorded on cottons. Lower values indicate proprioceptive inhibition from dental contact;

ATTIV compares how dental contacts affect temporal versus masseter muscles. It assesses sagittal contact prevalence and the mandible’s lever arm. A positive value indicates dominant masseter activity, while a negative value indicates dominant temporal activity (anterior centre of gravity). Healthy subjects typically range between +10% and −10%.

Both the MVC analysis [34] and the specific indices used in the previous study by Pittar et al. [26] have been reported as reproducible in the literature.

### 2.5. Experimental Protocol

**Figure 1:**
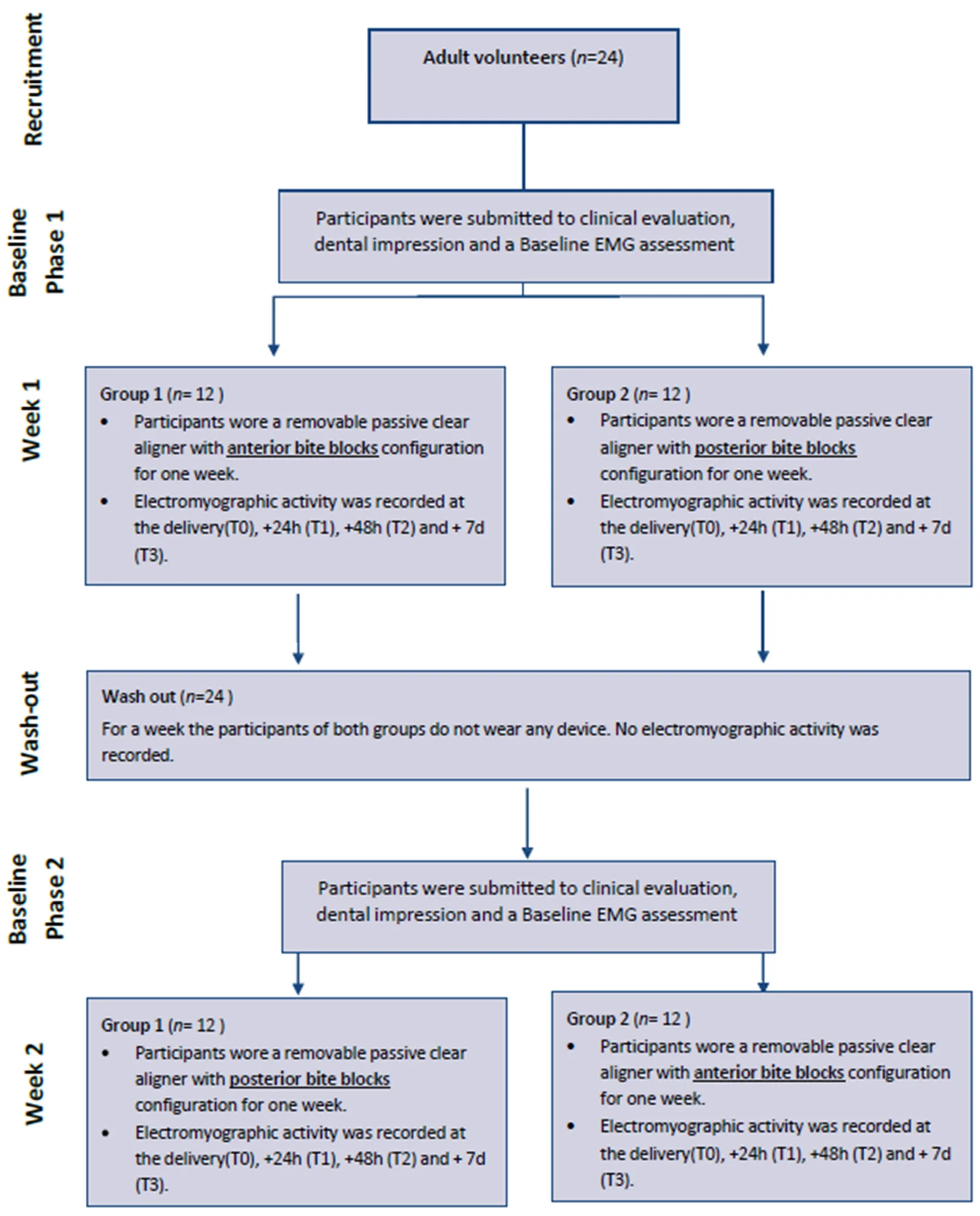
Flow chart of the study protocol. Arrows indicate the chronological sequence of study phases. On delivery day (T0), initial EMG assessments were taken without aligners. After device delivery and fit verification by an experienced operator, EMG was repeated. Participants wore splints > 20 h daily. EMG assessments were repeated at 24 h (T1), 48 h (T2) and 7 days (T3), with each recording being made with and without BBs.

The experimental protocol comprised two phases preceded by baseline repeatability checks. Figure 1 summarises the timing and phases of the protocol. At Phase 1 baseline, scans and EMG assessments were performed and psychological questionnaires completed. Participants were randomised into two groups receiving either Configuration 1 or 2 devices.

Following one week, devices were discontinued for a 1-week washout, with final EMG confirming repeatability. The procedure was then repeated with groups crossing over to the alternate configuration.

### 2.6. Statistical Analysis

Outliers were identified via interquartile ranges. Normally distributed data were analysed using means and standard deviations with Levene’s test for variability; non-normal data used medians, interquartile ranges and Fligner–Killeen tests. Significant variability changes prompted unsupervised clustering (GMM, spectral, hierarchical, K-means) to detect subgroups. Student’s *t*-tests compared psychosocial scores between clusters. A *p*-value < 0.05 was considered statistically significant. Analyses employed Microsoft Excel 365 and R version 4.0.0.

## 3. Results

Baseline repeatability was verified using paired *t*-tests comparing initial and T0 measurements without appliances (IMPACT, *p* = 0.45; ATTIV, *p* = 0.34).

Overall, an increase in the inter-individual variability of muscular work intensity was observed with anterior bite blocks but not with posterior ones.

Table 1 shows electromyographic indices for anterior BBs. After excluding four outliers, data were normally distributed (Shapiro–Wilk). The ATTIV index shifted from −15.21% (WO) to −10.48% (T0-W), indicating variable temporalis–masseter balance. IMPACT decreased from 76.10% (WO) to 46.70% (T0-W), recovering to 68.45% at T3. Levene’s test showed significantly increased IMPACT variability at T1 (*p* = 0.030) and T3 (*p* = 0.047), but no ATTIV changes (Table 2).

Table 3 presents posterior BB electromyographic parameters. ATTIV shifted from −18.3% (WO) to −11.76% (T0-W), showing reduced anterior temporalis dominance. IMPACT declined from 73.5% (WO) to 54% (T0-W), fluctuating to 57.5% at T3. Non-parametric analysis was used due to non-normal distribution. Table 4 shows no significant variability differences for either index (Fligner–Killeen test). Since statistically significant differences were found only when anterior BB were introduced, only these results were further analysed with cluster analysis.

**Figure 2:**
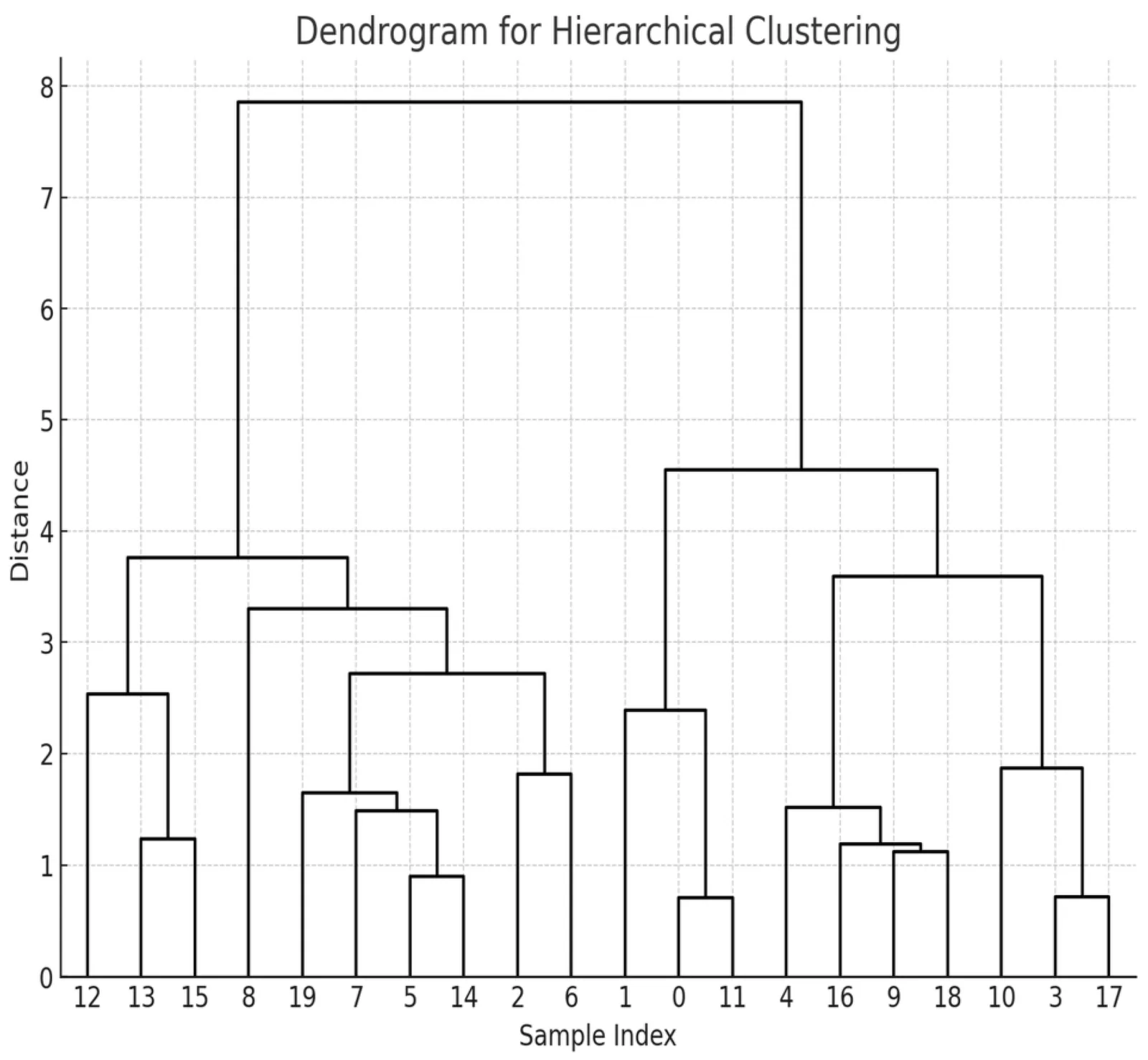
Dendrogram created using hierarchical clustering for the anterior BB. Each cluster is composed of 10 subjects.

Table 5 shows standard deviations from four clustering methods for IMPACT variability with anterior BBs. All methods reduced variability versus original data (IMP_T1W SD = 30.13%; IMP_T3W SD = 30.49%). Hierarchical clustering achieved the lowest SDs (T1 = 16.12%; T3 = 24.81%) and was selected for further analysis. Non-significant Levene’s tests (*p* > 0.05) versus IMP_T0_WO SD (19.77%) confirmed cluster homogeneity. Figure 2 displays the corresponding dendrogram.

**Figure 3:**
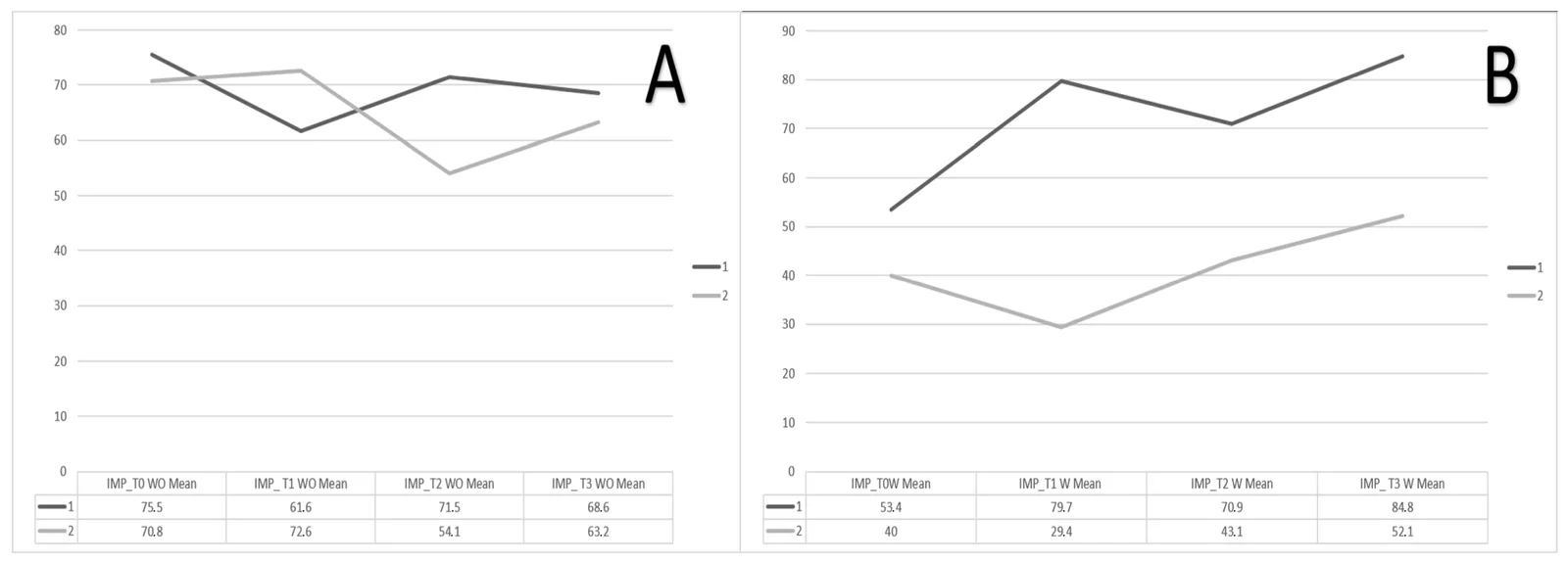
IMPACT parameter calculated without (**A**) and with (**B**) the anterior BB. The two clusters were identified through hierarchical clustering.

Figure 3 compares mean IMPACT values over time in two clusters identified by hierarchical clustering. Without the anterior BB, both clusters exhibit similar baseline IMPACT activity. With the anterior BB, Cluster 1 shows an initial drop in IMPACT followed by recovery, indicating quick adaptation, whereas Cluster 2 demonstrates a persistent reduction in IMPACT over time, reflecting prolonged neuromuscular inhibition. This figure highlights the distinct temporal patterns of muscular response between the subgroups wearing anterior BBs.

Table 6 shows *t*-test results for questionnaire scores between anterior BB clusters. SSAS scores differed significantly (mean difference = 4.0; *p* = 0.040), with Cluster 1 reporting higher values (9.6 ± 5.62) than Cluster 2 (5.6 ± 1.07). OBC scores showed no significant difference (*p* = 0.633), suggesting somatosensory amplification influences muscle response variability to anterior BBs.

**Figure 4:**
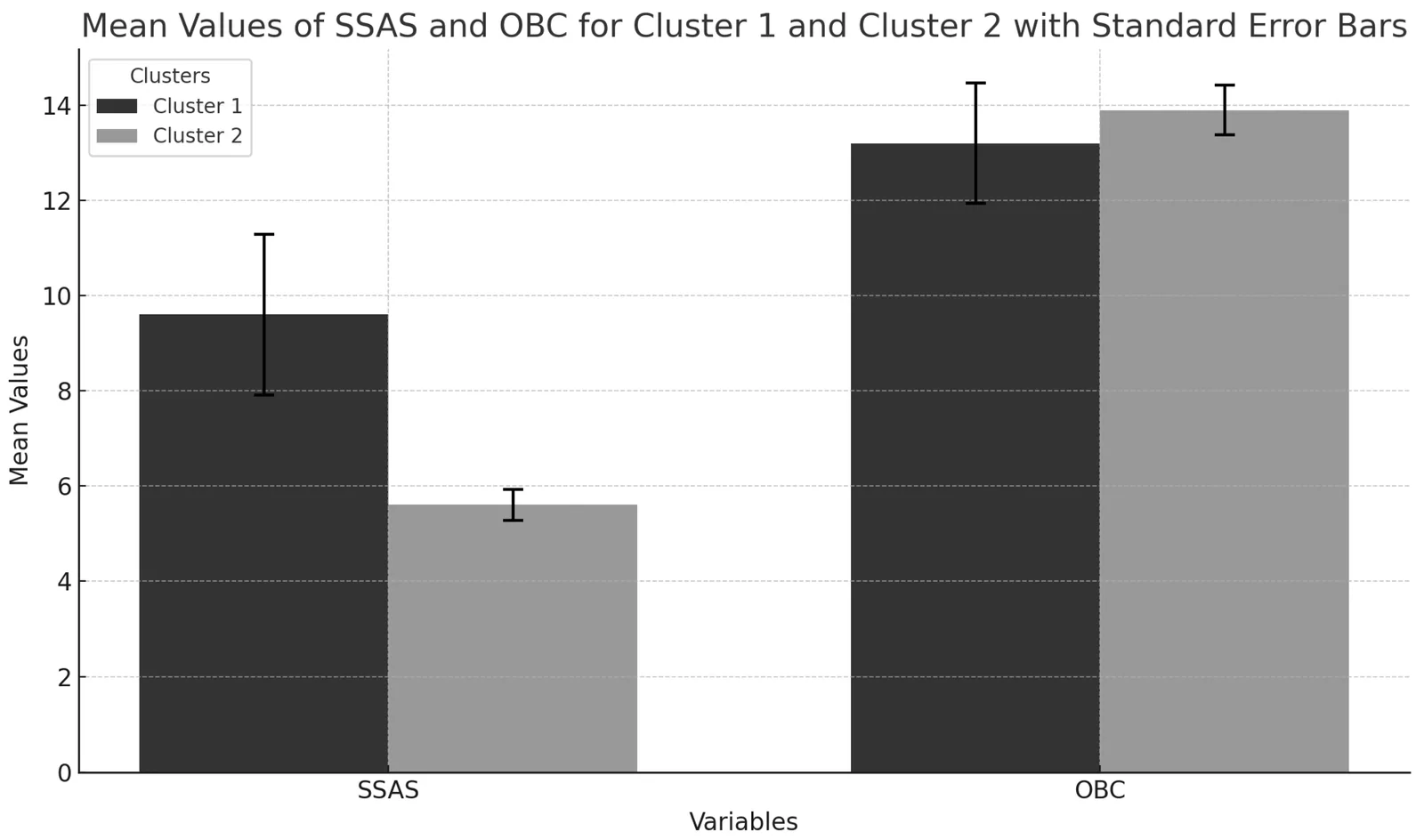
Bar chart for questionnaire results divided into the two clusters. Mean values are reported as raw numbers. Bars indicate standard error of the mean.

Figure 4 presents bar charts of mean scores for the SSAS and OBC, separated by the two clusters identified in the anterior BB analysis.

## 4. Discussion

This study analysed how masticatory muscles respond to bite blocks (BBs) on clear aligners. Anterior BBs caused significant and variable neuromuscular changes, while posterior BBs did not.

Two response patterns emerged: both groups showed reduced masticatory effort immediately after BB placement. However, Cluster 1 recovered to baseline within 24 h and increased further by T3, whereas Cluster 2 showed a progressive decline. These differences were only apparent with the BBs in place, and Cluster 1 reported greater somatosensory sensitivity.

Studies analysing BBs used in traditional orthodontics have reported similar results [22]. One study found that anterior BBs transiently reduced EMG activity during clenching. However, muscle activity generally returned to baseline within one to three months, with no significant effects [16]. Pativetpinyo and colleagues [17] investigated the electromyographic changes in masticatory muscles following the application of orthodontic band cement on upper molars to raise the bite. While the authors did not specifically analyse changes in variability, their data suggest an immediate increase in variability at the time of delivery. Our posterior BB findings align, showing no significant variability changes overall but an initial IMPACT variability increase at T0. This contrasts with anterior BBs, where variability emerged within 24 h, indicating a later onset of inter-individual variability for posterior interventions.

To analyse the effects of clear aligners, two systematic reviews were conducted [29,30]. Lekavičiūtė and colleagues [30] reported no statistically significant differences. The authors observed results that were very close to significance, including a reduction in masseter functionality followed by a return to normal activity and increased temporalis muscle activity. Patients reported in the review [30] were treated without BBs and underwent active treatment. These are important differences compared with our protocol. The use of clear aligners results in modifications to patients’ occlusal contacts. These modifications vary between patients, primarily depending on their initial occlusal state and material thickness. The studies included in the systematic reviews [29,30] did not examine the position of occlusal contacts after patients received their aligners. Findings from our study, particularly the differences in muscular adaptation between anterior and posterior occlusal precontacts, suggest that this factor could explain the variability observed in the authors’ results.

The clinical utility of BBs requires discussion. While posterior BBs are intended to facilitate molar intrusion through clenching [8], our findings seem to show homogeneous muscular reduction across patients, potentially explaining their limited efficacy. Conversely, anterior BBs aim to achieve molar extrusion for curve of Spee levelling [9], but our data suggests this mechanism may only benefit patients with sustained muscular work reduction. Those who increase musculoskeletal effort may overcome the anterior BBs due to material flexibility.

BB thickness represents another source of variability. While fixed in our study, clinical thickness varies. Manns et al. [14] reported greater muscular responses with larger elevations, though variability findings conflict between studies [12,15]. Unlike Lou et al. [27], who found psychological correlations, we identified SSAS differences between clusters, suggesting somatosensory amplification influences muscular adaptation variability in healthy subjects.

The present findings should also be interpreted in light of previous research investigating psychological and behavioural factors in orthodontic populations. In a previous study by Cioffi et al. [35], somatosensory amplification and trait-related psychological constructs were shown to be associated with variability in orthodontic pain perception during tooth movement induced by elastic separators. That study highlighted that individuals with higher somatosensory amplification tend to report greater discomfort and exhibit increased attentional focus on oral sensations during orthodontic procedures.

In the present investigation, similar psychological constructs were assessed using the SSAS and the Oral Behaviour Checklist (OBC) to explore whether trait-related somatosensory amplification and oral behavioural patterns could also be associated with variability in masticatory muscle response to occlusal alterations induced by passive aligners with bite blocks. Although the outcome measures differ (pain perception versus neuromuscular activity), both studies support the concept that inter-individual variability in orthodontic responses may be partially influenced by psychological and behavioural traits.

Previous research [36] has demonstrated that somatosensory amplification and anxiety are associated with altered tactile perception within the stomatognathic system. They reported that individuals with higher levels of somatosensory amplification and anxiety exhibit a reduced ability to accurately detect minimal interdental thicknesses, suggesting that psychological traits may influence the processing of occlusal sensory input rather than simply enhancing sensory sensitivity.

These findings provide a potential mechanistic explanation for the role of SSAS in the present study. If somatosensory amplification affects the accuracy and processing of periodontal and oral tactile information, it may also influence how individuals perceive and adapt to experimentally induced occlusal modifications such as bite blocks. This altered sensory processing could, in turn, modulate the afferent input to the central nervous system and consequently affect the neuromuscular response of the masticatory system recorded via sEMG.

A preliminary investigation preceded the data analysis presented in this study to assess the reproducibility of the measurements. The maximum voluntary contraction measurements, normalised according to the technique described by Ferrario et al. [10], were found to be reproducible across both phases of the research. The findings reported here are consistent with those of Suvinen et al. [34], which also emphasise the reproducibility of MVC measurements. The healthy reference range for IMPACT is generally reported as 80–120% [10], yet our baseline values (75–76%) consistently fell below this threshold. It is important to note that our participants were recruited as healthy volunteers based on the absence of clinically detectable pain or temporomandibular disorders, rather than on the basis of normative EMG values. It is plausible that the reference range of 80–120% was originally established in populations with different demographic or occlusal characteristics, and that our sample, despite being asymptomatic, may exhibit physiological variations in resting muscle activity that are still within a functional range. Previous studies employing similar sEMG protocols have reported comparable baseline values in healthy young adults [22,34], suggesting that this finding may reflect normal physiological variability rather than a pathological deviation. Nevertheless, we acknowledge that the discrepancy between our baseline IMPACT values and the reported reference range represents a limitation, as it may affect the generalizability of our findings to populations with different baseline muscle activity profiles. Future studies should consider stratifying participants based on baseline EMG characteristics to further elucidate the clinical significance of this observation. Several limitations must be acknowledged. Despite reproducible baselines, the anterior BB data contained four outliers, and the posterior data were non-normally distributed. The small sample size and non-parametric tests may have increased false-negative risk. Limiting clustering to two groups potentially overlooked additional subgroups. Volunteer recruitment prevented cephalometric selection, and healthy participants limit generalizability to TMD populations. In addition, the fact that the heterogeneity of orthodontic anomalies did not serve as a primary stratification variable represents a significant limitation. The lack of sex balance in our sample (75% female) may have influenced our findings, and it could represent a potential confounder that was not controlled for in the present study.

## 5. Conclusions

Within the limitations of the study, in the same participants, wearing passive aligners with anterior bite blocks significantly increased the inter-individual variability of muscular work intensity relative to the no-appliance baseline, whereas passive aligners with posterior bite blocks produced no significant changes.

Hierarchical clustering identified two subgroups when Anterior BB were tested:

Cluster 1 showed a reduction in muscular work intensity at baseline, returning to regular activity within 24 h.

Cluster 2 showed a prolonged reduction in muscular work intensity, with further decreased activity at the 24 h follow-up.

SSAS scores differed significantly between clusters, suggesting that somatosensory amplification could influence neuromuscular responses, but further research is needed. Given the small, predominantly female sample and the exploratory nature of the clustering, these findings should be interpreted with caution and confirmed in larger studies.

## Figures and Tables

**Table 1:** Mean and standard deviation (St.dev) for all the parameters analysed. Without anterior bite block: WO. With anterior bite block: W. T0: bite block adaptation and delivery; T1: 24 h from T0; T2: 48 h from T0; T3: 7 days from T0. Mean and standard deviation are expressed as percentage.

	ATTIV Baseline WO	ATTIV T0 WO	ATTIV T0 W	ATTIV T1 W	ATTIV T2 W	ATTIV T3 W	IMPBaseline WO	IMP T0 WO	IMP T0 W	IMP T1 W	IMP T2 W	IMP T3 W
Mean	−14.93	−15.21	−10.48	−17.03	−15.79	−12.49	75.21	76.10	46.70	54.55	57.00	68.45
St.dev	21.13	20.84	29.37	26.16	22.82	22.64	22.57	20.60	19.77	30.13	22.91	30.49

**Table 2:** Levene’s test results. Without anterior bite block: WO. With anterior bite block: W. T0: bite block adaptation and delivery; T1: 24 h from T0; T2: 48 h from T0; T3: 7 days from T0. * Indicates results that are statistically significant (*p* < 0.05).

Comparison	Levene’s Statistic	*p*-Value
ATTIV_T0 WO vs. ATTIV_T0 W	0.013	0.909
ATTIV_T0 WO vs. ATTIV_T1 W	0.264	0.609
ATTIV_T0 WO vs. ATTIV_T2 W	0.006	0.940
ATTIV_T0 WO vs. ATTIV_T3 W	1.136	0.292
IMP_T0 WO vs. IMP_T0 W	0.101	0.753
IMP_T0 WO vs. IMP_T1 W	5.081	0.030 *
IMP_T0 WO vs. IMP_T2 W	0.830	0.368
IMP_T0 WO vs. IMP_T3 W	4.233	0.047 *

**Table 3:** Median and interquartile range (IQR) for parameters calculated with posterior bite blocks. Without posterior bite block: WO. With posterior bite block: W. T0: bite block adaptation and delivery; T1: 24 h from T0; T2: 48 h from T0; T3: 7 days from T0. All measures are expressed as percentage.

	ATTIV T0 WO	ATTIV T0 W	ATTIV T1 W	ATTIV T2 W	ATTIV T3 W	IMP T0 WO	IMP T0 W	IMP T1 W	IMP T2 W	IMP T3 W
Median	−18.30	−11.76	−7.81	−9.02	−4.94	73.50	54.00	69.50	66.50	57.50
IQR	24.46	27.65	20.10	23.12	23.28	33.25	49.00	40.75	46.50	19.75

**Table 4:** Fligner–Killeen test results for IQR comparison; statistics and *p*-value are reported. Without posterior bite block: WO. With posterior bite block: W. T0: bite block adaptation and delivery; T1: 24 h from T0; T2: 48 h from T0; T3: 7 days from T0. No statistically significant difference is reported.

Variable	Fligner–Killeen Stat	*p*-Value
ATTIV_T0 WO vs. ATTIV_T0 W	<0.01	0.936
ATTIV_T0 WO vs. ATTIV_T1 W	0.163	0.686
ATTIV_T0 WO vs. ATTIV_T2 W	0.034	0.855
ATTIV_T0 WO vs. ATTIV_T3 W	0.728	0.394
IMP_T0 WO vs. IMP_T0 W	0.220	0.638
IMP_T0 WO vs. IMP_T1 W	1.340	0.247
IMP_T0 WO vs. IMP_T2 W	0.640	0.423
IMP_T0 WO vs. IMP_T3 W	0.070	0.789

**Table 5:** Standard deviation obtained with the four different clustering techniques. With anterior bite block: W. All standard deviations are expressed as percentage.

	Original	GMM Cluster 1	GMM Cluster 2	Spectral Cluster 1	Spectral Cluster 2	Hierarchical Cluster 1	Hierarchical Cluster 2	K-Means Cluster 1	K-Means Cluster 2
IMP_T0W	19.77	19.95	14.00	14.00	19.95	21.95	15.60	19.95	14.00
IMP_T1 W	30.13	18.93	12.89	12.89	18.93	16.12	15.84	18.93	12.89
IMP_T2 W	22.91	13.81	13.11	13.11	13.81	14.97	21.33	13.81	13.11
IMP_T3 W	30.49	30.69	28.35	28.35	30.69	24.81	27.43	30.69	28.35

**Table 6:** T-test and *p*-value for questionnaire scores between the two clusters. * Indicates statistically significant difference.

	SSAS	OBC
**Group 1**		
Mean	9.6	13.2
St.dev	5.62	4.21
**Group 2**		
Mean	5.6	13.9
St.dev	1.07	1.73
**Differences**		
Mean	4.0	−0.7
St.error	1.81	1.44
**T.stat**	2.210	−0.486
***p*.value**	0.040 *	0.633

## Data Availability

The data presented in this study are available on request from the corresponding author due to privacy/ethical restrictions.

## Abbreviations

The following abbreviations are used in this manuscript:

Abbreviation	Definition
BB	Bite Block
PCA	Passive Clear Aligner
sEMG	Surface Electromyography
MVC	Maximum Voluntary Clenching
COT	Maximum Voluntary Clenching with Cotton Roll Interposition
SSAS	Somatosensory Amplification Scale
OBC	Oral Behaviour Checklist
TMD	Temporomandibular Disorders
GMM	Gaussian Mixture Model
IQR	Interquartile Range
